# Supplementary material for: Comparison of the Therapeutic Effects of Adipose- and Bone Marrow-Derived Mesenchymal Stem Cells on Renal Fibrosis
Source: Int J Mol Sci. 2023 Nov 29;24(23):16920. doi: 10.3390/ijms242316920 (PMC10706978; doi:10.3390/ijms242316920)
Supplement: Supplementary file 1 [file ijms-24-16920-s001.zip › ijms-2719387-supplementary.pdf]

Supplemental material

Comparison of the therapeutic effects of adipose- and bone marrow-derived mesenchymal stem cells on renal fibrosis

Maria Yoshida[1], Ayumu Nakashima[1,2*], Naoki Ishiuchi[1,2], Kisho Miyasako[1], Keisuke Morimoto[1], Yoshiki Tanaka[1], Kensuke Sasaki[1], Satoshi Maeda[2,3] and Takao Masaki[1*]

[1]Department of Nephrology, Hiroshima University Hospital, 1-2-3 Kasumi, Minami-ku, Hiroshima, Hiroshima 734-8551, Japan

[2]Department of Stem Cell Biology and Medicine, Graduate School of Biomedical & Health Sciences, Hiroshima University, 1-2-3 Kasumi, Minami-ku, Hiroshima, Hiroshima 734-8553, Japan

[3]TWOCELLS Company, Limited, 16-35 Hijiyama-honmachi, Minami-ku, Hiroshima, Hiroshima 732-0816, Japan.

This file includes supplementary figure S1.

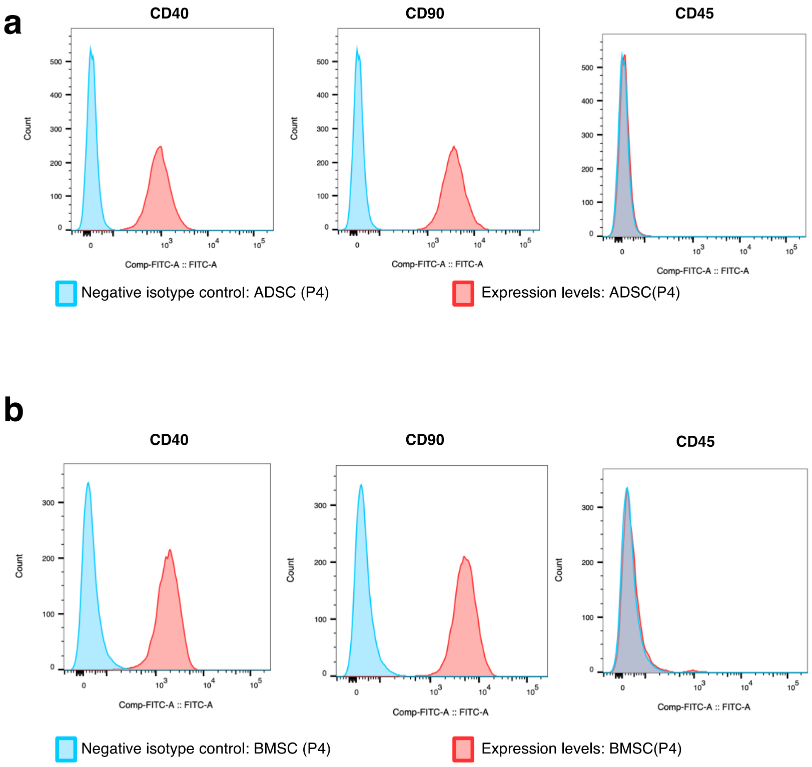

Supplementary figure S1. Cell surface markers on mesenchymal stem cells (MSCs). Flow cytometry showed the expression of surface markers on (**a**) adipose tissue-derived MSCs (ADSCs) and (**b**) bone marrow-derived MSCs (BMSCs).
